# Provision and Perception of Physiotherapy in the Nonoperative Management of Degenerative Cervical Myelopathy (DCM): A Cross-Sectional Questionnaire of People Living With DCM

**DOI:** 10.1177/2192568220961357

**Published:** 2020-10-01

**Authors:** Max B. Butler, Oliver D. Mowforth, Abdul Badran, Michelle Starkey, Timothy Boerger, Iwan Sadler, Julia Tabrah, Caroline Treanor, Lucy Cameron Grad Dip Phys, Sukhvinder Kalsi-Ryan, Rodney J. Laing, Benjamin M. Davies, Mark R. N. Kotter

**Affiliations:** 1 University of Cambridge, Cambridge, UK; 2 Myelopathy.org, Cambridge, UK; 3 KITE Research Institute, UHN, Toronto, Ontario, Canada; 4 NHS Berkshire Healthcare, Berkshire, UK; 5 Beaumont Hospital, Dublin, Republic of Ireland; * Joint senior authors

**Keywords:** myelopathy, spondylosis, cervical, degenerative, neuro, spinal cord injury

## Abstract

**Study Design:**

Cross-sectional survey.

**Objectives:**

Degenerative cervical myelopathy (DCM) is a common syndrome of acquired spinal cord impairment caused by canal stenosis secondary to arthritic changes of the spine. International guidelines consider physiotherapy an option for mild, stable DCM; however, few studies have been conducted on nonoperative management. The objective was to determine current usage and perceptions of nonoperative physiotherapy for DCM.

**Methods:**

Persons with DCM were recruited to a web-based survey. Participants with complete responses that had not received surgery were included (n = 167). Variables included symptom duration, treatment history, current disability, and demographic characteristics.

**Results:**

Disease and demographic characteristics were equivalent between those who did and did not receive physiotherapy. In all, 19.5% of physiotherapy recipients reported subjective benefit from physiotherapy. Those perceiving benefit had significantly higher mJOA (modified Japanese Orthopaedic Association) scores, lower neck pain scores, and shorter symptom duration. In multivariate logistic regression analysis, those with mild DCM were more likely to perceive benefit than those with severe DCM, as were those with moderate DCM (to a lesser extent). Individuals whose diagnosis was delayed 1 to 2 years were less likely to perceive benefit than those that waited 0 to 6 months.

**Conclusions:**

The provision of nonoperative physiotherapy in the management of DCM is inconsistent and appears to differ from international guidelines. Few patients perceived benefit from physiotherapy; however, this was more likely in those with mild DCM and in those with shorter symptom durations. Further work is needed to establish the appropriate role of physiotherapy for this population.

## Introduction

Degenerative cervical myelopathy (DCM) is a progressive neurological condition, characterized by symptomatic cervical cord compression secondary to age-related changes in the cervical spine.^1^ DCM is the commonest spinal cord disorder worldwide,^1^ with up to 5% prevalence estimated in individuals aged older than 40 years. Given an aging population, an increase in prevalence and disease burden from DCM is anticipated.

The cervical spinal cord processes and transmits information between the brain and the body. Abnormal function of the spinal cord causes diverse symptoms including pain, paresthesia, weakness, unsteadiness, frequent falls, loss of dexterity and incontinence.^2^ This substantially impacts quality of life; one recent study found people with DCM have among the worst health-related quality of life (Short Form–36 [SF-36]) scores of any chronic disease.^3^

Surgical decompression is the only evidence-based treatment demonstrated to halt DCM progression and offer worthwhile but often incomplete recovery.^4,5^ The natural history of DCM is poorly understood and the rate of symptom progression in an individual is highly variable and difficult to predict. In people with mild DCM, the risks of surgery may outweigh any benefit. For these individuals, physiotherapy may have a role^6^ with limited data suggesting that outcomes from nonoperative management may be comparable to surgery.^7^ Consequently, recent international guidelines only recommended surgery for progressive, moderate, or severe disease. Conservative management, including physiotherapy, is recommended for mild, stable forms of the condition. Although it is unlikely to alter degenerative changes, physiotherapy may improve neck conditioning; pain reduction; monitoring of progression, and disease education.

Guidelines are key for knowledge translation, and their dissemination is continuing. These recommendations are typically based on clinical studies designed by health care professionals, which often overlook the perspective of people with DCM.^8-10^ The aim of this study was to survey people with cervical myelopathy to determine if they had received physiotherapy and their perceptions of that treatment. We did not capture the intent of the physiotherapy or expectations of treatment.

## Methods

The survey was designed and is reported following the Checklist for Reporting Results of Internet E-Surveys.^11^

### Survey Design

An online survey was designed using SurveyMonkey (Survey Monkey), Facebook (Facebook), Twitter (Twitter), Google AdWords (Google), and Myelopathy.org, a UK-registered charity, with a large online, international community of people with DCM. The website is a hub for support groups, educational resources, and information about DCM research.

The survey questions be found in Supplementary Material 1. Questions assessed disease time course, treatment history, current disability, and respondent demographics. Respondents were asked if they had received physiotherapy and, if so, whether they found it helpful. Current disability was assessed in part through the patient-derived modified Japanese Orthopaedic Association (p-mJOA) score. The mJOA is a composite score based on upper and lower limb motor function, upper limb sensation and sphincter function, that is widely used to assess myelopathy severity.^12^ It is fully validated for this purpose, including when self-reported.^13^

Data was stored on password-protected computers. The sequence of questions and order of responses was the same for all respondents.

### Ethical Approval and Informed Consent

The study was ethically approved by a university ethics committee and was performed in accordance with the relevant guidelines and regulations.

All respondents completed the questionnaire voluntarily and were informed before doing so that their responses would be used anonymously for research purposes. The initial page stated the study objectives and host organization details. This acted as electronic consent and continuation was taken as agreement. No respondent-identifiable information was stored.

### Participants

Respondents with DCM who had not received surgery at the time of the survey were included. Those that had received surgery were excluded.

### Recruitment

The recruitment process has been described previously.^14^ An open survey design was used. People with DCM were recruited to an online questionnaire administered by SurveyMonkey. Social media posts, supported by Myelopathy.org, recruited participants, alongside advertisements implemented with Google AdWords. Respondents were not contacted outside the survey.

### Administration

A link to the survey was hosted on a landing page on Myelopathy.org. The survey was not administered via email. Completion was voluntary, and no incentives were offered. Responses were collected from October 2015 to August 2017. A total of 42 survey items were distributed over 15 survey pages. Responses with incomplete answers to medical management questions were excluded. Missing data analysis was performed to evaluate potential bias from excluded responses (Supplementary Material 2). Respondents were able to review their answers before survey submission.

### Response Rates

Google Analytics, a Web-based analytics service, was used to measure the number of visitors to Myelopathy.org. Survey view rate was 6.3% (1663/26 501), participation rate was 67.0% (1114/1663), and completion rate for the overall survey was 69.8% (778/1114) (Supplementary Material 3).

### Preventing Multiple Entries From the Same Individual

Duplicate responses were limited by respondent IP addresses.

### Statistical Analysis

Descriptive analyses are reported as means ± standard deviations for continuous variables and frequencies and percentages for categorical variables, unless otherwise specified.

Univariate analyses were performed to compare those who received physiotherapy with those who did not, and those who perceived benefit from physiotherapy with those who did not. The chi-square test of homogeneity was used for categorical variables that met minimum expected count requirements; Fisher’s exact test was used for those that did not. The Mann-Whitney *U* test was used for respondent age.

A multivariate logistic regression was performed to control for potential confounding variables. Variables with a *P* < .10 in univariate analysis were included in multivariate analysis. Multivariate regression was not performed to analyze receipt of physiotherapy, as no variables had a *P* < .10 in univariate analysis. Multivariate regression was performed to analyze perceived benefit from physiotherapy; the assumption of no multicollinearity was met and the Hosmer and Lemeshow goodness of fit test indicated that the model was a good fit. Four standardized residuals had values of >2 standard deviations; these were retained in the analysis. The final model statistically significantly predicted the dependent variable over and above the intercept-only model, χ^2^(14) = 29.470, *P* = .009. The ability of the model to predict perceived benefit from physiotherapy was assessed by receiver operating characteristic (ROC) curve analysis.

Analyses were conducted using SPSS Statistics software, version 26 (IBM Corporation). Significance was set at *P* < .05.

## Results

**Figure 1.**
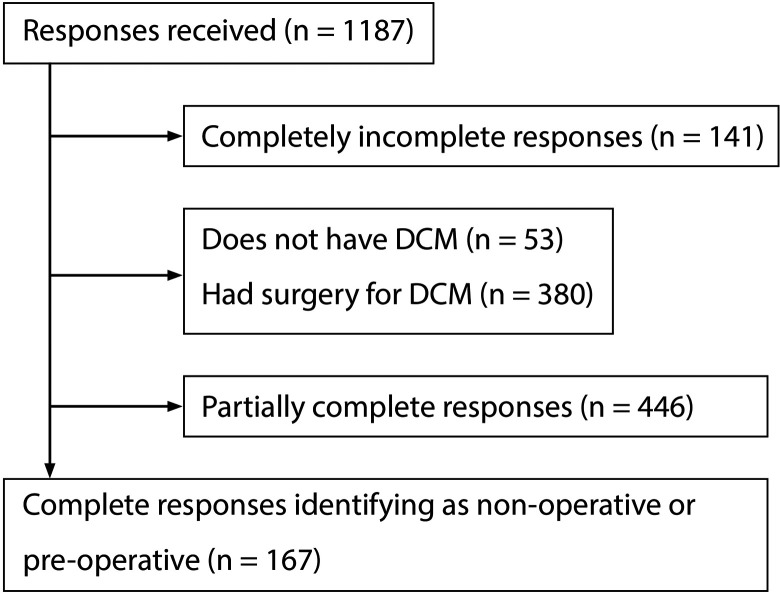
Flow diagram of response selection.

Participants, with complete survey responses, that had not received surgery (n = 167) were included in this analysis (Figure 1). Mean age was 54 years, and over two-thirds of respondents were female (120/167, 72.3%). Most respondents (Table 1) were from the United Kingdom (86/167, 51.5%) or the United States (56/167 33.5%), and were White/Caucasian (147/166, 88.6%). Approximately half of respondents were employed, either full-time (53/167, 31.7%) or part-time (27/167, 16.2%). Almost one-third stated being unable to work due to disability (47/167, 28.1%).

**Table 1.** Cohort Demographics (n = 167).

Variable	n	%
Gender	166	
Female	120	72.3
Male	46	27.7
Age, years, mean (SD)	53.9	(10.8)
Country of current residence	167	
United Kingdom	86	51.5
United States	56	33.5
Canada	11	6.6
Australia	3	1.8
Other	11	6.6
Ethnicity	166	
White/Caucasian	147	88.6
Asian	9	5.4
Hispanic	6	3.6
Black or African American	2	1.2
Mixed	1	0.6
Prefer not to answer	1	0.6
Employment status	167	
Employed, full-time	53	31.7
Employed, part-time	27	16.2
Disabled, not able to work	47	28.1
Unemployed, looking for work	5	3
Unemployed, not looking for work	7	4.2
Retired	28	16.8
Annual income, UK£	165	
0-9999	14	8.5
10 000-24 999	24	14.5
25 000-49 999	41	24.8
50 000-99 999	24	14.5
100 000-149 999	5	3
150 000+	5	3
Prefer not to answer	52	31.5
Education level	164	
Less than high school degree	18	11
High school degree or equivalent (eg, GED)	30	18.3
Some college but no degree	33	20.1
Associate degree	16	9.8
Bachelor’s degree	31	18.9
Graduate degree	36	22

### Physiotherapy Provision/Usage Did Not Predict Severity of Disability

A total of 49% of respondents had received physiotherapy (82/167). There was no significant difference in disability between those who did and did not receive physiotherapy (Nurick 1.9 ± 1.4 vs 2.0 ± 1.4, *P* = .850; mJOA, *P* = .311, Table 2). In total, 79.3% of individuals who received physiotherapy had moderate or severe DCM, compared with 70.6% of individuals who did not receive physiotherapy. There were no significant differences in time to diagnosis, duration of symptoms, pain scores or demographic measures between those who did and did not receive physiotherapy.

**Table 2.** Physiotherapy Usage, Between-Group Comparison (n = 167).

Variable	Received physiotherapy:		*P*
	Yes	No	
Sample size, n	82	85	—
Gender: female, n (%)	63 (77.8)	57 (67.1)	.123^a^
Age of respondent, years, mean	53.5	56	.683^b^
Dependence on others to support daily activities, n (%)	5 (47.2)	29 (34.1)	.255^b^
Time to diagnosis, n (%)	82	85	.120^b^
0-6 months	18 (22.0)	27 (31.8)	
7-12 months	9 (11.0)	15 (17.6)	
1-2 years	23 (28.0)	12 (14.1)	
2-5 years	23 (28.0)	20 (23.5)	
>5 years	9 (11.0)	11 (12.9)	
Duration of symptoms, n (%)	82	85	.465^b^
0-1 year	15 (18.3)	18 (21.2)	
2-3 years	32 (39.0)	22 (25.9)	
3-10 years	23 (28.0)	32 (37.6)	
10-25 years	11 (13.4)	12 (14.1)	
>25 years	1 (1.2)	1 (1.2)	
Nurick score, mean ± SD	1.9 ±1.4	2.0 ±1.4	.850^b^
mJOA score, mean ± SD	11.9 ±3.1	12.8 ±2.9	.311^b,d^
≤11, n (%)	34 (41.5)	27 (31.8)	
12-14, n (%)	31 (37.8)	33 (38.8)	
≥15, n (%)	17 (20.7)	25 (29.4)	
Current neck pain score, mean	5.4	4.9	.811^b^
Best neck pain score, mean	3.9	3.6	.781^c^
Worst neck pain score, mean	7.4	6.9	.487^c^
Education level, n (%)	80	84	.258^b^
Less than high school degree	6 (7.5)	12 (14.3)	
High school degree or equivalent	15 (18.8)	15 (17.9)	
Some college but no degree	17 (21.3)	16 (19)	
Associate degree	7 (8.8)	9 (10.7)	
Bachelor’s degree	15 (18.8)	16 (19)	
Graduate degree	20 (25)	16 (19)	
Ethnicity, n (%)	82	84	.915^c^
White/Caucasian	72 (87.8)	75 (89.3)	
Black or African American	3 (3.7)	3 (3.6)	
Asian	6 (7.3)	3 (3.6)	
Other	1 (1.2)	3 (3.6)	
Country of residence, n (%)	82	80	.615^b^
United Kingdom	46 (56.1)	40 (50)	
United States	23 (28)	33 (41.3)	
Canada	6 (7.3)	5 (6.3)	
Australia	1 (1.2)	2 (2.5)	
Other	6 (7.3)	0 (0)	

Abbreviation: mJOA, modified Japanese Orthopaedic Association.

^a^ Mann-Whitney *U* test.

^b^ Chi-square test.

^c^ Fisher’s exact test.

^d^ For this analysis, scores were grouped into ≤11 (severe), 12-14 (moderate), ≥15 (mild).

### Perceived Benefit of Physiotherapy Is Greater in Those With Mild DCM

**Figure 2.**
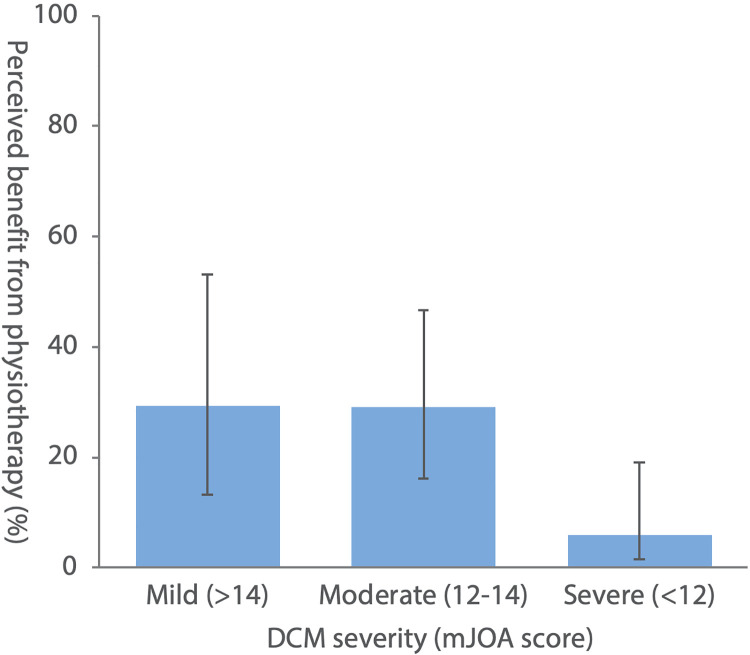
Perceived benefit of physiotherapy (%) versus degenerative cervical myelopathy (DCM) severity (modified Japanese Orthopaedic Association [mJOA] score). Error bars represent 95% binomial confidence intervals.

One-fifth of physiotherapy recipients stated a subjective benefit (16/82, 19.5%) of physiotherapy. A total of 29.4% (5/17) of respondents with mild DCM (mJOA ≥15) perceived benefit from physiotherapy, compared with 29.0% (9/31) of respondents with moderate DCM (mJOA 12-14), and 5.9% (2/34) of respondents with severe DCM (mJOA ≤12, Figure 2). Those perceiving benefit from physiotherapy had significantly higher mJOA scores than those that did not perceive benefit (*P* = .032, Table 3). There was no significant difference in Nurick score between those who perceived benefit and those who did not (1.6 ± 1.4 vs 2.0 ± 1.4; *P* = .398). Current neck pain scores were significantly lower in those who perceived benefit (3.9 ± 2.9 vs 5.7 ± 2.4, *P* = .031); however, there was no significant difference between groups for best and worst neck pain scores. Respondents perceiving benefit had shorter symptom duration (*P* = .049) than those not perceiving benefit. There was no significant difference in time to diagnosis, dependence on others, or demographic measures, except for ethnicity (*P* = .042).

**Table 3.** Perceived Benefit of Physiotherapy: Univariate Analysis (n = 82).

Variable	Benefit perceived?		*P*
	Yes	No	
Sample size, n	16	66	
Gender: female, n (%)	11 (68.8)	52 (78.8)	.733^a^
Age of respondent, mean	53.9	53.9	.693^c^
Dependence on others to support daily activities, n (%)	4 (25.0)	31 (47.0)	.111^b^
Time to diagnosis, n (%)	16	66	.055^c^
0-6 months	8 (50)	10 (15.2)	
7-12 months	1 (6.3)	8 (12.1)	
1-2 years	2 (12.5)	21 (31.8)	
2-5 years	3 (18.8)	20 (30.3)	
>5 years	2 (12.5)	7 (10.6)	
Duration of symptoms, n (%)	16	66	.049^c^
0-1 year	6 (37.5)	9 (13.6)	
2-3 years	3 (18.8)	29 (43.9)	
3-10 years	3 (18.8)	20 (30.3)	
10-25 years	4 (25)	7 (10.6)	
>25 years	0 (0)	1 (1.5)	
Nurick score, mean ± SD	1.6 ±1.4	2.0 ±1.4	.398^c^
mJOA score, mean ± SD	13.7 ±2.2	11.4 ±3.2	.032^b,d^
≤11, n (%)	2 (12.5)	32 (48.5)	
12-14, n (%)	9 (56.3)	22 (33.3)	
≥15, n (%)	5 (31.3)	12 (18.2)	
Current neck pain score, mean	3.9	5.7	.031^c^
Best neck pain score, mean	2.6	4.2	.218^c^
Worst neck pain score, mean	6.4	7.7	.297^c^
Education level, n (%)	15	65	.960^c^
Less than high school degree	2 (13.3)	4 (6.2)	
High school degree or equivalent	2 (13.3)	13 (20)	
Some college but no degree	1 (6.7)	16 (24.6)	
Associate degree	0 (0)	7 (10.8)	
Bachelor’s degree	4 (26.7)	11 (16.9)	
Graduate degree	6 (40)	14 (21.5)	
Ethnicity, n (%)	16	66	.042^c^
White/Caucasian	12 (75)	60 (90.9)	
Black or African American	0 (0)	3 (4.5)	
Asian	4 (25)	2 (3)	
Other	0 (0)	1 (1.5)	
Country of residence, n (%)	13	63	.325^c^
United Kingdom	7 (53.8)	39 (61.9)	
United States	5 (38.5)	18 (28.6)	
Canada	1 (7.7)	5 (7.9)	
Australia	0 (0)	1 (1.6)	
Other	0 (0)	0 (0)	

Abbreviation: mJOA, modified Japanese Orthopaedic Association.

^a^ Mann-Whitney *U* test.

^b^ Chi-square test.

^c^ Fisher’s exact test.

^d^ For this analysis, scores were grouped into ≤11 (severe), 12-14 (moderate), ≥15 (mild)

In multivariate logistic regression analysis, disease severity (classified by mJOA score^5^) was independently associated with perceived benefit from physiotherapy (Table 4). Those with mild DCM (mJOA >14) were more likely to perceive benefit from physiotherapy than those with severe DCM (mJOA <12; OR = 28.5, 95% CI = 2.0-410.8, *P* = .014). Those with moderate DCM (mJOA 12-14) were also more likely to perceive benefit from physiotherapy than those with severe DCM, however, to a lesser extent (OR = 12.7, 95% CI = 1.3-126.2, *P* = .030). Individuals who waited 1 to 2 years to receive a diagnosis of DCM were less likely to perceive benefit from physiotherapy than those that waited 0 to 6 months (OR = 0.04, 95% CI = <0.01-0.8, *P* = .035). Respondent ethnicity and symptom duration did not significantly predict perceived benefit from physiotherapy. Receiver operating characteristic (ROC) curve analysis showed the area under the ROC curve (AUC) was 0.866, reflecting excellent predictive performance of the model.^15^

**Table 4.** Perceived Benefit of Physiotherapy: Multivariate Analysis (n = 82).

Variable	*P*	OR	95% CI for OR
Ethnicity			
White/Caucasian	—	—	—
Asian	.066	14.41	0.84–247.68
Black or African American	.999	<0.01	<0.01 to <0.01
Other	.302	12.1	0.11–1381.38
mJOA score			
≤11	—	—	—
12-14	.030*	12.72	1.28–126.19
≥15	.014*	28.51	1.98–410.38
Time to diagnosis			
0-6 months	—	—	—
6-12 months	.124	0.07	<0.01–2.04
1-2 years	.035*	0.04	<0.01–0.80
2-5 years	.075	0.08	<0.01–1.29
>5 years	.148	0.09	<0.01–2.33
Duration of symptoms			
0-1 year	—	—	—
2-3 years	.836	1.3	0.11–16.24
3-10 years	.851	1.33	0.07–26.06
10-25 years	.065	18.43	0.84–405.94
>25 years	1	<0.01	<0.01 to <0.01
Current neck pain (1-10)	.522	0.04	0.79–1.61

Abbreviation: mJOA, modified Japanese Orthopaedic Association.

*P < .05.

## Discussion

This is the first study to survey the provision and perception of physiotherapy in the non-operative management of DCM. The majority of recipients of physiotherapy had moderate or severe DCM. A minority of respondents perceived benefit from physiotherapy. Perceived benefit was greatest in milder forms of the disease or in respondents with shorter symptom duration. One-third of those with mild or moderate DCM perceived benefit, compared to less than 10% of those with severe DCM. Respondents were most likely to perceive benefit if diagnosed within 6 months of the symptom onset.

### Physiotherapy Provision Is Inconsistent and Does Not Align With International Guidelines

Our analysis found no difference in disease characteristics between those who received and did not receive physiotherapy. International guidelines recommend nonoperative physiotherapy as an option for mild, stable DCM.^5^ Most physiotherapy recipients in this survey had moderate or severe disease, for whom surgery is recommended. As the international guidelines were released during this survey, this should be considered preimplementation practice. People with DCM can self-refer to physiotherapy in certain jurisdictions, hence the physiotherapy community should be an important target for knowledge translation of international guidelines; it is important that those with moderate or severe DCM understand the limitations of physiotherapy and the importance of early surgical referral. Nevertheless, physiotherapy appears to be underused in cases where it is indicated; most respondents with mild DCM had not received physiotherapy.

### Few Perceive Benefit From Physiotherapy, but Perception of Benefit Is More Likely in Those With Milder DCM and Shorter Symptom Duration

Overall, a minority of respondents perceived benefit from physiotherapy (20%). This proportion was greatest in those with mild DCM (30%), and lowest in those with severe DCM (6%). Multivariate analysis found participants with mild DCM were 13 times more likely to perceive benefit than those with moderate DCM, and 29 times more likely than those with severe DCM. The evidence for conservative management of mild DCM is limited.^7,16^ These findings support reserving physiotherapy for milder disease presentations.

Previous work has shown that delays to DCM diagnosis are common.^17^ Delays are associated with greater disease severity^18^ and limit surgical outcomes.^1^ In this study, those with delayed diagnosis were less likely to perceive benefit from physiotherapy, independent of their disease severity.

The natural history of DCM is poorly understood and represents a research priority as identified by AO Spine RECODE-DCM.^9,19^ While unpredictable in rate, emerging evidence indicates that DCM is progressive, even in milder clinically stable forms. Using laboratory gait analysis,^20^ Kalsi-Ryan et al^21^ have demonstrated subclinical progression in mild DCM over 1 to 2 years. Additionally, using quantitative magnetic resonance imaging, Martin et al^22^ identified subclinical evidence of DCM. As physiotherapy aims to reduce symptoms and physical impairment rather than modifying the disease process, these findings may align with the trend for early only benefit to physiotherapy.

### Limitations

The survey was conducted through Myelopathy.org. Respondents were given a description of DCM its symptoms and asked if they had been diagnosed by a medical professional; it is possible that some respondents did not have DCM. To limit recall bias, time-related questions used categorical ranges, and there was no survey time limit.

Most survey respondents were female; however, gender is not known to be a risk factor for DCM or to affect its prognosis.^23,24^ No differences in perception and provision of physiotherapy between genders were observed in this study. Though minority ethnic groups had a small representation, the breakdown (88.6% White, 1.2% Black, 5.4% Asian), is comparable to the UK population (86% White, 3.3% Black, 7.5% Asian).^25^ Multivariate analysis was used, minimizing bias due to demographic characteristics.

Missing data analysis showed that with one exception, missing data did not introduce statistically significant bias (Supplementary Material 2). Respondents with incomplete answers had a longer time to diagnosis than those with complete answers. Multivariate analysis minimized the impact of introduced bias on other variables; it is unlikely that absent responses have affected our findings.

Respondents were asked if they had received physiotherapy or surgery, but not whether they had been offered it; some may have been offered therapy but declined or not yet received it, and individuals declining surgery may have been offered physiotherapy to minimize impairment.

This survey was retrospective; participants were asked if they had perceived benefit from physiotherapy but disability scores (eg, mJOA and Nurick) were contemporary. This temporal difference may have overestimated the proportion of people with moderate and severe DCM receiving physiotherapy and may have led to recall bias. The use of multivariate analysis, including symptom duration, has attempted to mitigate this.

Finally, the logistic regression odds ratios have broad confidence intervals. This reflects small samples sizes due to low rates of perceived physiotherapy benefit. Our conclusions remain valid when using values at the interval extremes.

### Future Directions

The role of physiotherapy was identified as an important research uncertainty during the AO Spine RECODE-DCM James Lind Alliance research priority setting partnership. The findings here align with the published literature: there is likely a group who benefit. Clearer identification of this subgroup and the physiotherapy parameters (type and dosage) that deliver the most benefit will be key to ensuring better outcomes, alongside the dissemination of recommendations to clinical practice.

New approaches to DCM assessment will be important to optimize management. Many current assessments lack the sensitivity to detect or track small changes in disease severity, particularly in early and mild forms of the disease. Improved sensitivity would enable earlier detection of patients who have progressed from mild to moderate DCM, or who have progressive DCM (ie, have moved into the zone of surgical intervention being recommended by the AO Spine guidelines).^19,26^ The development of new clinical assessments for DCM is a further AO Spine RECODE-DCM research priority.^19^

Other emergent techniques, such as the use of microstructural magnetic resonance imaging, serological biomarkers, and genetic analysis, may enhance hospital-based assessment and bring the benefits of personalized medicine to the diagnosis and management of DCM.^27^

## Conclusions

The provision physiotherapy in the management of DCM is inconsistent and differs from the recommendations of international guidelines. Few people with cervical myelopathy perceive benefit from physiotherapy but the greatest perceived benefit was found in respondents with mild DCM. Further work is needed to establish the appropriate role of physiotherapy for this population.

## Supplemental Material

sj-pdf-1-gsj-10.1177_2192568220961357 – Supplemental Material for Provision and Perception of Physiotherapy in the Nonoperative Management of Degenerative Cervical Myelopathy (DCM): A Cross-Sectional Questionnaire of People Living With DCM Click here for additional data file. Supplemental Material, sj-pdf-1-gsj-10.1177_2192568220961357 for Provision and Perception of Physiotherapy in the Nonoperative Management of Degenerative Cervical Myelopathy (DCM): A Cross-Sectional Questionnaire of People Living With DCM by Max B. Butler, Oliver D. Mowforth, Abdul Badran, Michelle Starkey, Timothy Boerger, Iwan Sadler, Julia Tabrah, Caroline Treanor, Lucy Cameron Grad Dip Phys, Sukhvinder Kalsi-Ryan, Rodney J. Laing, Benjamin M. Davies and Mark R. N. Kotter in Global Spine Journal

sj-pdf-2-gsj-10.1177_2192568220961357 – Supplemental Material for Provision and Perception of Physiotherapy in the Nonoperative Management of Degenerative Cervical Myelopathy (DCM): A Cross-Sectional Questionnaire of People Living With DCM Click here for additional data file. Supplemental Material, sj-pdf-2-gsj-10.1177_2192568220961357 for Provision and Perception of Physiotherapy in the Nonoperative Management of Degenerative Cervical Myelopathy (DCM): A Cross-Sectional Questionnaire of People Living With DCM by Max B. Butler, Oliver D. Mowforth, Abdul Badran, Michelle Starkey, Timothy Boerger, Iwan Sadler, Julia Tabrah, Caroline Treanor, Lucy Cameron Grad Dip Phys, Sukhvinder Kalsi-Ryan, Rodney J. Laing, Benjamin M. Davies and Mark R. N. Kotter in Global Spine Journal

sj-pdf-3-gsj-10.1177_2192568220961357 – Supplemental Material for Provision and Perception of Physiotherapy in the Nonoperative Management of Degenerative Cervical Myelopathy (DCM): A Cross-Sectional Questionnaire of People Living With DCM Click here for additional data file. Supplemental Material, sj-pdf-3-gsj-10.1177_2192568220961357 for Provision and Perception of Physiotherapy in the Nonoperative Management of Degenerative Cervical Myelopathy (DCM): A Cross-Sectional Questionnaire of People Living With DCM by Max B. Butler, Oliver D. Mowforth, Abdul Badran, Michelle Starkey, Timothy Boerger, Iwan Sadler, Julia Tabrah, Caroline Treanor, Lucy Cameron Grad Dip Phys, Sukhvinder Kalsi-Ryan, Rodney J. Laing, Benjamin M. Davies and Mark R. N. Kotter in Global Spine Journal
