# Supplementary material for: Provision and Perception of Physiotherapy in the Nonoperative Management of Degenerative Cervical Myelopathy (DCM): A Cross-Sectional Questionnaire of People Living With DCM
Source: Global Spine J. 2020 Oct 1;12(4):638–45. doi: 10.1177/2192568220961357 (PMC9109573; doi:10.1177/2192568220961357)
Supplement: sj-pdf-1-gsj-10.1177_2192568220961357 – Supplemental Material for Provision and Perception of Physiotherapy in the Nonoperative Management of Degenerative Cervical Myelopathy (DCM): A Cross-Sectional Questionnaire of People Living With DCM [file sj-pdf-1-gsj-10.1177_2192568220961357.pdf]

Supplemental Digital Content 1. Example Questionnaire.

How did you find this survey?
- Via a link on Google
- Twitter
- Facebook
- Prompted by your healthcare provider
- Other (please specify)

Do you suffer from cervical myelopathy?
- Yes
- No

Are you a carer for a patient with cervical myelopathy?
- Yes
- No

How long did it take to be diagnosed with cervical myelopathy?
- 0-6 months
- 7-12 month
- 1-2 years
- 2-5 years
- >5 years

How long have you suffered with cervical myelopathy?
- 0-1 year
- 2-3 years
- 3-10 years
- 10-25 years
- > 25 years

Have you undergone physiotherapy for cervical myelopathy? Was it helpful?
- Yes, and it was helpful
- Yes, but it wasn't helpful
- No

Have you undergone surgery for cervical myelopathy?
- Yes
- No

How many times have you undergone surgery?
- Open-Ended Response

What type of surgery have you undergone (tick all that apply)
- Anterior (eg ACDF)
- Posterior (eg Laminectomy or Laminoplasty)
- Multilevel
- Instrumented (You have metalwork in your neck)
- Other (please specify)

How long since your last operation (number of months) approximately?
- Open-Ended Response

How long did it take to recover from the operation (number of months) approximately?

- Open-Ended Response

How effective was the surgery?

- Good – significant improvement; some minimal or intermittent symptoms persist
- Fair – minimal improvement
- Poor – Symptoms unchanged or worse

Did you undergo post-operative physiotherapy as part of your treatment? Was it helpful?

- Yes, and it was helpful
- Yes, but it wasn't helpful
- No

In general, how would you rate your overall health?

- Very good
- Good
- Fair
- Poor

How does this compare to last year?

- Much better
- A little better
- Much the same
- A little worse
- Much worse

The following questions are about activities you might do during a typical day. Does your health now limit you in these activities? If so, how much?

Options for each row: Yes, limited a lot; Yes, limited a little; No, not limited at all

- Vigorous activities, such as running, lifting heavy objects, participating in strenuous sports
- Moderate activities, such as moving a table, pushing a vacuum cleaner, bowling, or playing golf
- Lifting or carrying groceries
- Climbing several flights of stairs
- Climbing one flight of stairs
- Bending, kneeling, or stooping
- Walking more than a mile
- Walking several hundred yards
- Walking one hundred yards
- Bathing or dressing yourself

Are you dependent on another (or others) to support you in your daily activities?

- Yes
- No

During the past 4 weeks, how much of the time have you had any of the following problems with your work or other regular daily activities as a result of your physical health?

Options for each row: All of the time; Most of the time; Some of the time; A little of the time; None

- Cut down on the amount of time you spent on work or other activities
- Accomplished less than you would like
- Were limited in the kind of work or other activities
- Had difficulty performing the work or other activities (for example, it took extra effort)

During the past 4 weeks, how much of the time have you had any of the following problems with your work or other regular daily activities as a result of any emotional problems (such as feeling depressed or anxious)?
Options for each row: All of the time; Most of the time; Some of the time; A little of the time; None

- Cut down on the amount of time you spent on work or other activities
- Accomplished less than you would like
- Did work or activities less carefully than usual

During the past 4 weeks, to what extent has your physical health or emotional problems interfered with your normal social activities with family, friends, neighbors, or groups?

- Extremely
- Quite a bit
- Moderately
- Slightly
- Not at all

How much bodily pain have you had during the past 4 weeks?

- Very severe
- Severe
- Moderate
- Mild
- None

During the past 4 weeks, how much did pain interfere with your normal work (including both work outside the home and housework)?

- Extremely
- Quite a bit
- Moderately
- Slightly
- Not at all

Please indicate the intensity of the current, best and worst pain affecting your NECK over the past 24h on a scale of 0 (no pain) to 10 (worst pain imaginable)

- Currently
- Best
- Worst

Please indicate the intensity of the current, best and worst pain affecting your ARMS/HANDS over the past 24h on a scale of 0 (no pain) to 10 (worst pain imaginable)

- Currently
- Best
- Worst

How do your symptoms affect your everyday life and ability to walk?

- I suffer from symptoms such as pain and numbness, but not imbalance, incontinence or weakness
- I suffer imbalance, incontinence or weakness, but my walking is unaffected.
- I have a slight difficulty walking, but it does not prevent me from working or completing my daily activities
- I have moderate difficulty walking, which prevents me from working or completing daily activities.
- I require assistance to walk
- I am unable to walk

These questions are about how you feel and how things have been with you during the past 4 weeks. For each question, please give the one answer that comes closest to the way you have been feeling. How much of the time during the past 4 weeks...

Options for each row: All of the time; Most of the time; Some of the time; A little of the time; None

- Did you feel full of life?
- Have you been very nervous?
- Have you felt so down in the dumps that nothing could cheer you up?
- Have you felt calm and peaceful?
- Did you have a lot of energy?
- Have you felt downhearted and depressed?
- Did you feel worn out?
- Have you been happy?
- Did you feel tired?

During the past 4 weeks, how much of the time has your physical health or emotional problems interfered with your social activities (like visiting friends, relatives, etc.)?

- All of the time
- Most of the time
- Some of the time
- A little of the time
- None

How TRUE or FALSE is each of the following statements for you?

Options for each row: Definitely true; Mostly true; Mostly false; Definitely false; Don't know

- I seem to get sick a little easier than other people
- I am as healthy as anybody I know
- I expect my health to get worse
- My health is excellent

How does cervical myelopathy affect your hands?

- I am unable to move my hands at all
- I can move my hands a little, but I am unable to feed myself
- I can feed myself, but I am unable to button up my shirt
- I can button my shirt, but I find it very difficult and it takes a long time
- I can button my shirt with only slight difficulty
- My hands work normally

How does cervical myelopathy affect your legs?

Response

- I am paralysed and unable to move or feel my legs
- I can feel my legs but I am unable to move them at all
- I can move my legs, but I cannot walk
- I can walk but I require a walking aid
- I am a little unsteady on my feet, but I walk up and down the stairs without holding the hand rail
- I can walk up and down stairs, but I have to hold the hand rail
- I am a little unsteady, but I can walk unaided
- My legs are unaffected

How does cervical myelopathy affect your hands?

Response

- I am unable to feel my hands
- I have pain or severe loss of feeling (incl. numbness, tingling) in my arms or hands
- I have mild loss of feeling (including numbness, tingling) in my hands
- I have no loss of feeling in my arms or hands

How does cervical myelopathy affect your bladder?

Responseå

- I can’t control when I pass urine
- I have marked difficulty passing urine
- I have mild difficulty passing urine
- I have no difficulty passing urine

The consequences of spinal cord injury are classified into 7 different categories by researchers. For you as a patient, what are the research priorities for you (please rank where 1 is the most important and 7 is the least important)? What would you like researchers to focus on?

- Elimination of Pain
- Arm/Hand Function
- Walking Function
- Bladder/Bowel Function
- Sexual Function
- Upper Body/Trunk Function
- Normal Sensation

Are you male or female?

- Male
- Female

How old are you?

- Open-Ended Response

In what country do you currently reside?

- United Kingdom
- United States
- Australia
- Other (please specify)

What is your ethnicity? (Tick all that apply)

- Black or African American
- White/Caucasian
- Asian
- Prefer not to answer
- Other (please specify)

How much total combined money did all members of your HOUSEHOLD earn last year?

- £0 to £9,999
- £10,000 to £24,999
- £25,000 to £49,999
- £50,000 to £99,999
- £100,000 to £149,999
- £150,000+
- Prefer not to answer

Which of the following categories best describes your employment status?

- Employed, working full-time
- Employed, working part-time
- Not employed, looking for work
- Not employed, NOT looking for work
- Retired
- Disabled, not able to work

What is the highest level of school you have completed or the highest degree you have received?

- Graduate degree
- Bachelor degree
- Associate degree
- Some college but no degree
- High school degree or equivalent (e.g., GED)
- Less than high school degree
