# Supplementary material for: Provision and Perception of Physiotherapy in the Nonoperative Management of Degenerative Cervical Myelopathy (DCM): A Cross-Sectional Questionnaire of People Living With DCM
Source: Global Spine J. 2020 Oct 1;12(4):638–45. doi: 10.1177/2192568220961357 (PMC9109573; doi:10.1177/2192568220961357)
Supplement: sj-pdf-2-gsj-10.1177_2192568220961357 – Supplemental Material for Provision and Perception of Physiotherapy in the Nonoperative Management of Degenerative Cervical Myelopathy (DCM): A Cross-Sectional Questionnaire of People Living With DCM [file sj-pdf-2-gsj-10.1177_2192568220961357.pdf]

Variable	Complete data (n=167) %	Mean (SD)	n	Incomplete data (n = 514) %	Mean (SD)	n	p	Test
**Gender:**						362	0.63	X^2
Female	72.3	-	120	75.1	-	272		
Male	27.7	-	46	33.1	-	90		
**Age of respondent:**		53.9(10.8)	164		55.4(10.0)	193	0.782	MWW
**Country of current residence:**			167			293	0.535	Fisher's
UK	51.5	-	86	49.5	-	145		
USA	33.5	-	56	37.2	-	109		
Canada	6.6	-	11	5.5	-	16		
Australia	1.8	-	3	3.8	-	11		
Other	6.6	-	11	4.1	-	12		
**Ethnicity:**			166			283	0.324	Fisher's
White / Caucasian	88.6	-	147	91.2	-	258		
Asian	5.4	-	9	2.8	-	8		
Black or African American	1.2	-	2	3.5	-	10		
Other	4.8	-	8	2.5	-	7		
**Employment status:**			167			292	0.688	X^2
Employed, full-time	31.7	-	53	27.1	-	79		
Employed, part-time	16.2	-	27	11.6	-	34		
Disabled, not able to work	28.1	-	47	30.5	-	89		
Unemployed, looking for work	3	-	5	3.4	-	10		
Unemployed, not looking for work	4.2	-	7	7.5	-	22		
Retired	16.8	-	28	19.9	-	58		
**Annual income:**			165			286	0.761	X^2
£0 to £9,999	8.5	-	14	9.8	-	28		
£10,000 to £24,999	14.5	-	24	13.3	-	38		
£25,000 to £49,999	24.8	-	41	16.8	-	48		
£50,000 to £99,999	14.5	-	24	14.0	-	40		
£100,000 to £149,999	3	-	5	6.3	-	18		
£150,000+	3	-	5	3.1	-	9		
Prefer not to answer	31.5	-	52	36.7	-	105		
**Education level:**			164			289	0.197	X^2
Less than high school degree	11	-	18	9.7	-	28		
High school degree or equivalent	18.3	-	30	16.3	-	47		
Some college but no degree	20.1	-	33	30.4	-	88		
Associate degree	9.8	-	16	9.7	-	28		
Bachelor degree	18.9	-	31	19.0	-	55		
Graduate degree	22	-	36	14.9	-	43		
**Dependent on others to support daily activities**			167			410	0.868	X^2
Yes	38.3	-	64	36.8	-	151		
No	61.7	-	103	63.2	-	259		
**Time to diagnosis**			164			446	0.042	X^2
0-6 months	27.4	-	45	29.6	-	132		
7-12 month	14.6	-	24	13.5	-	60		
1-2 years	20.7	-	34	17.5	-	78		
2-5 years	25.0	-	41	14.1	-	63		
>5 years	12.2	-	20	25.3	-	113		
**Duration of symptoms**			165			446	0.311	X^2
0-1 year	20.0	-	33	21.3	-	95		
1-3 years	32.1	-	53	26.9	-	120		
3-10 years	32.7	-	54	29.4	-	131		
10-25 years	13.9	-	23	17.7	-	79		
> 25 years	1.2	-	2	4.7	-	21		
**Nurick score, mean (SD)**		1.95 (1.40)	167		1.81(1.52)		0.889	X^2
**mJOA score, mean (SD)**		12.34 (3.05)	167		12.72 (2.99)			
**mJOA score, classed**			167			255	0.71	X^2
Severe (0-11)	36.5		61	36.9		94		
Moderate (12-14)	38.3		64	32.2		82		
Mild (>14)	25.1		42	31.0		79		
**Neck pain score: current, mean (SD)**		5.11 (2.50)	167		5.42 (2.69)	350	0.348	X^2
**Neck pain score: best, mean (SD)**		3.74 (2.14)	167		4.01 (2.32)	350	0.974	Fisher's
**Neck pain score: worst, mean (SD)**		7.19 (2.58)	167		7.13 (2.79)	350	0.321	Fisher's
