# Supplementary material for: Provision and Perception of Physiotherapy in the Nonoperative Management of Degenerative Cervical Myelopathy (DCM): A Cross-Sectional Questionnaire of People Living With DCM
Source: Global Spine J. 2020 Oct 1;12(4):638–45. doi: 10.1177/2192568220961357 (PMC9109573; doi:10.1177/2192568220961357)
Supplement: sj-pdf-3-gsj-10.1177_2192568220961357 – Supplemental Material for Provision and Perception of Physiotherapy in the Nonoperative Management of Degenerative Cervical Myelopathy (DCM): A Cross-Sectional Questionnaire of People Living With DCM [file sj-pdf-3-gsj-10.1177_2192568220961357.pdf]

<u>**Response Data Calculations**</u>

All definitions are copied from the Checklist for Reporting Results of Internet E-Surveys (CHERRIES).

<table>
<tr><th>Rate</th><th>Definition</th><th>Variables</th><th>Calculation</th></tr>
<tr><td rowspan="2">View rate</td><td rowspan="2">Unique survey visitors
÷
Unique site visitors</td><td>Unique site visitors
• Myelopathy UK unique site visitors in October 2015 to August 2017 = 26,501</td><td rowspan="2">6.3%
(1663/26501)</td></tr>
<tr><td rowspan="2">Unique survey visitors
• SurveyMonkey completion rate = 67% (individuals that visited the survey ÷ individuals that submitted a response).
• 1114 people submitted a response from October 2015 to August 2017
• Therefore 1663 people visited the survey in that time</td></tr>
<tr><td rowspan="2">Participation rate</td><td rowspan="2">Unique visitors who agreed to participate
÷
Unique survey visitors</td><td rowspan="2">67.0%
(1114/1663)</td></tr>
<tr><td rowspan="2">Users who agreed to participate
• Submission of the survey, regardless of quantity of data entered, to presumed to indicate agreement to participate
• 1114 people submitted a response from October 2015 to August 2017</td></tr>
<tr><td rowspan="2">Completion rate</td><td rowspan="2">Ratio of users who finished the survey/users who agreed to participate</td><td rowspan="2">69.8%
(778/1114)</td></tr>
<tr><td>Users who completed the survey
• Extracted data was divided into:
  o Completely incomplete (n = 283)
  o No DCM (n = 53)
  o Completely complete (n = 778)
• All respondents that did not have DCM did not answer any questions beyond their disease status, therefore were judged to be incomplete answers
• Therefore 778 people completed the survey</td></tr>
</table>
